# Adapting Psychotherapy for Comorbid Substance use and Bipolar Disorder in Older Sexual Minorities: A Case Study

**DOI:** 10.1093/geroni/igab046.3556

**Published:** 2021-12-17

**Authors:** Sarah Nanami Morehouse, Ashley Stripling, Kirenia Brunson, Jodie Maccarrone, Jessica Choe, Julian Garcia, Nicholas Boston

**Affiliations:** 1 Nova Southeastern University, Davie, Florida, United States; 2 Nova Southeastern University, Fort Lauderdale, Florida, United States

## Abstract

Approximately 65 to 95% of individuals with bipolar disorder (BD) are diagnosed with an additional psychiatric condition (Kessler, 1999). Alcohol, the most commonly abused substance amongst individuals with BD (Xiao et al., 2016), has been linked to significant increases in suicide attempts, disability, hospitalizations, and mortality (Baldessarini et al., 2008; Goldberg et al., 1999; Mitchell et al., 2007; Nery & Soares, 2011). Despite these ill effects, little is known about how to effectively treat, or adapt existing treatment appropriately, for the growing numbers of individuals who are dually diagnosed with BD and alcohol use disorder (AUD) and hold the identity of lesbian, gay, bisexual, transgender, or queer (LGBTQ) in late life. Thus, the purpose of this study is to demonstrate how treatment was adapted to a self-identified gay man with comorbid BD and AUD from a relational, culturally sensitive perspective while simultaneously implementing two short-term interventions: cognitive behavioral therapy (CBT) and a behavioral substance use program. In line with Knight & Poon’s (2008) Contextual Life Span Theory for Adapting Psychotherapy with Older Adults (CALTAP) and a multicultural lens that incorporates relevant research on older LGBTQ individuals, modifications were made to the content, structure, language, and duration of therapy while cultivating a safe and empathic space. Idiographic data and progress monitoring measures suggests treatment resulted in substance use and distress reduction, as well as mood stabilization. However, additional booster sessions may be advantageous given the risk for substance abuse relapse and the compounding effect it may exert on persons with BD.

